# Multicenter Epidemiologic Study of Coronavirus Disease–Associated Mucormycosis, India

**DOI:** 10.3201/eid2709.210934

**Published:** 2021-09

**Authors:** Atul Patel, Ritesh Agarwal, Shivaprakash M. Rudramurthy, Manoj Shevkani, Immaculata Xess, Ratna Sharma, Jayanthi Savio, Nandini Sethuraman, Surabhi Madan, Prakash Shastri, Deepak Thangaraju, Rungmei Marak, Karuna Tadepalli, Pratik Savaj, Ayesha Sunavala, Neha Gupta, Tanu Singhal, Valliappan Muthu, Arunaloke Chakrabarti, MucoCovi Network

**Affiliations:** Sterling Hospital, Ahmedabad, India (A Patel);; Postgraduate Institute of Medical Education & Research, Chandigarh, India (R. Agarwal, S.M. Rudramurthy, V. Muthu, A. Chakrabarti);; Avaron Hospital, Ahmedabad (M. Shevkani);; All India Institute of Medical Science, New Delhi, India (I. Xess);; Apollo Hospital, Hyderabad, India (R. Sharma);; St. John’s Medical College, Bengaluru, India (J. Savio);; Apollo Hospital, Chennai, India (N. Sethuraman);; Care Institute of Medical Sciences, Ahmedabad (S. Madan);; Sir Ganga Ram Hospital, New Delhi (P. Shastri);; Kovai Medical Centre and Hospital, Coimbatore, India (D. Thangaraju);; Sanjay Gandhi Postgraduate Institute of Medical Sciences, Lucknow, India (R. Marak);; All India Institute of Medical Sciences, Bhopal, India (K. Tadepalli);; Venus Hospital, Surat, India (P. Savaj);; Hinduja Hospital, Mumbai, Maharashtra, India (A. Sunavala);; Medanta The Medicity, Gurgaon, India (N. Gupta);; Kokilaben Hospital, Mumbai, Maharashtra, India (T. Singhal)

**Keywords:** respiratory infections, severe acute respiratory syndrome coronavirus 2, SARS-CoV-2, SARS, COVID-19, coronavirus disease, zoonoses, viruses, coronavirus, fungi, mucormycosis, diabetes, epidemiology, amphotericin, posaconazole, isavuconazole, India

## Abstract

During September–December 2020, we conducted a multicenter retrospective study across India to evaluate epidemiology and outcomes among cases of coronavirus disease (COVID-19)–associated mucormycosis (CAM). Among 287 mucormycosis patients, 187 (65.2%) had CAM; CAM prevalence was 0.27% among hospitalized COVID-19 patients. We noted a 2.1-fold rise in mucormycosis during the study period compared with September–December 2019. Uncontrolled diabetes mellitus was the most common underlying disease among CAM and non-CAM patients. COVID-19 was the only underlying disease in 32.6% of CAM patients. COVID-19–related hypoxemia and improper glucocorticoid use independently were associated with CAM. The mucormycosis case-fatality rate at 12 weeks was 45.7% but was similar for CAM and non-CAM patients. Age, rhino-orbital-cerebral involvement, and intensive care unit admission were associated with increased mortality rates; sequential antifungal drug treatment improved mucormycosis survival. The COVID-19 pandemic has led to increases in mucormycosis in India, partly from inappropriate glucocorticoid use.

Secondary infections are known to complicate the clinical course of coronavirus disease (COVID-19). Bacterial infections are the most common secondary infections, but increasing reports of systemic fungal infections are causing concern. In the early part of the COVID-19 pandemic, <1% of secondary infections reported in COVID-19 patients were fungal (*1*,*2*). Preexisting conditions, indiscriminate use of antimicrobial and glucocorticoid drugs, and lapses in infection control practices are putative factors contributing to the emergence of systemic fungal infections in severe COVID-19 cases (*3*). After incidence of candidemia and invasive aspergillosis in COVID-19 patients increased (*4*,*5*), awareness of possible fungal co-infections increased among clinicians and microbiologists. One study reported invasive fungal infections in ≈6% of hospitalized COVID-19 patients (*6*). Occasional reports of COVID-19–associated mucormycosis (CAM) from various centers (*7*,*8*) and a series of 18 cases from a city in South India increased our concerns about CAM (*9*). 

India has a high burden of mucormycosis among patients with uncontrolled diabetes mellitus, and many severe COVID-19 patients have diabetes (*8*,*10*). India also is one of the countries worst affected by the COVID-19 pandemic. Thus, we would expect India to have many CAM cases. We conducted a nationwide multicenter study to evaluate the epidemiology and outcomes of CAM and compare the results with cases of mucormycosis unrelated to COVID-19 (non-CAM).

## Methods

### Study Design and Setting

**Figure 1:**
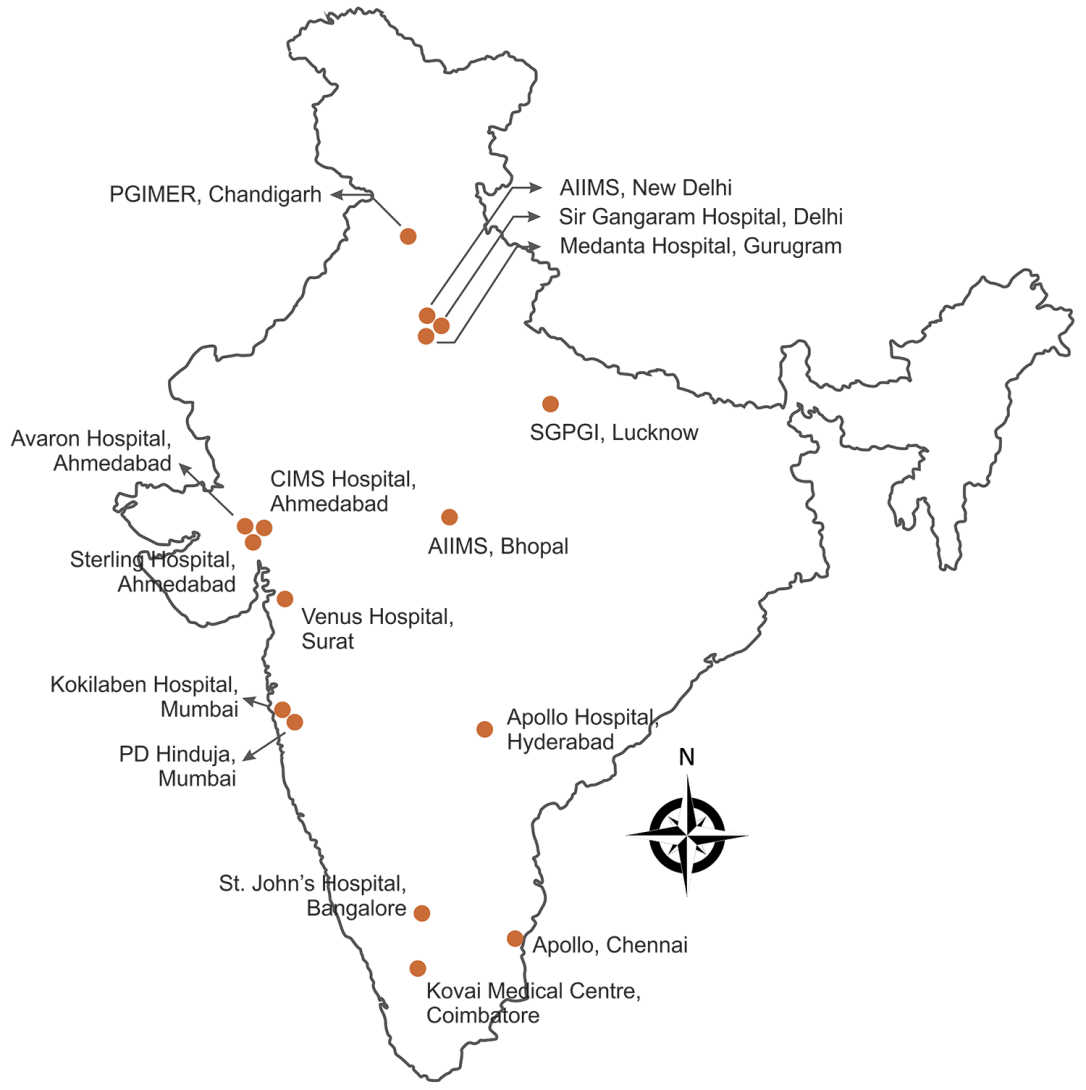
Locations of 16 healthcare centers participating in MucoCovi Network study on coronavirus disease–associated mucormycosis, India. AIIMS, All India Institute of Medical Sciences; CIMS, Care Institute of Medical Sciences; PD Hinduja, Parmanand Deepchand Hinduja; PGIMER, Post Graduate Institute of Medical Education & Research; SGPI, Sanjay Gandhi Postgraduate Institute

 We conducted a retrospective observational study involving 16 healthcare centers across India (Figure 1). We collected data for all confirmed mucormycosis cases among patients with and without COVID-19 reported during September 1–December 31, 2020. The ethics committees of the respective centers approved the study protocol.

### Study Subjects and Definitions 

We defined a case of mucormycosis as compatible clinical and radiologic manifestations and demonstration of fungi in the tissue or sterile body fluids of a patient by either direct microscopic visualization of broad ribbon-like aseptate hyphae or isolation of Mucorales. COVID-19 diagnosis was made in patients who tested positive for severe acute respiratory syndrome coronavirus 2 (SARS-CoV-2, the causative agent of COVID-19) RNA in respiratory specimens by reverse transcription PCR (RT-PCR) or a positive rapid antigen test. We defined CAM as the occurrence of proven mucormycosis in COVID-19 patients. 

Seven participating centers provided additional data on hospitalized COVID-19 patients and number of diagnosed CAM cases during the study period. The prevalence of CAM was calculated as the total number of CAM cases divided by the number of COVID-19 patients treated at the 7 participating centers during the study period. Similarly, the prevalence of CAM cases in the intensive care unit (ICU) was calculated as the total number of patients developing mucormycosis among COVID-19 patients who received treatment in the ICU. We classified CAM cases as early when mucormycosis was diagnosed <7 days after COVID-19 diagnosis and late when mucormycosis was diagnosed ≥8 days after COVID-19 diagnosis. We also collected the number of mucormycosis cases reported at the participating centers during the same months (September–December) of 2019. For patients who left the hospital against medical advice, we considered a worst-case scenario for mortality analysis and assumed the patients died.

### Study Procedure 

We developed a standard case-record form that we circulated to all the centers for data collection. We extracted the following information from the patient records: demographic characteristics; underlying diseases, such as diabetes mellitus, hematological malignancy, organ transplantation, and others; days to the diagnosis of mucormycosis before or after COVID-19 diagnosis; anatomic site of mucormycosis involvement; diagnostic modalities for mucormycosis, including microscopy, culture, or histopathology; treatment details, including antifungal drug therapy, surgical therapy, and other treatments; site of case management, including home, hospital ward, or ICU; immunosuppressive treatment received, such as glucocorticoid and other drugs; and outcome at 6 and 12 weeks. We classified multiple underlying diseases by using a hierarchical model. For instance, if a patient had hematologic malignancy and then diabetes mellitus developed due to the patient’s therapy, we considered hematologic malignancy as the primary risk factor. On the other hand, for patients with COVID-19 and preexisting uncontrolled diabetes, we regarded diabetes as the primary underlying disease.

### Treatment Details 

All patients received treatment for COVID-19 and mucormycosis according to protocol at the respective treating institution. We recorded the information regarding the type, dose, and duration of glucocorticoid drugs used for managing COVID-19, where available, by using dexamethasone-equivalent dose; 0.75 mg dexamethasone is equivalent to 4 mg methylprednisolone or 5 mg prednisolone. We classified glucocorticoid use as not indicated when any steroid was used for managing nonhypoxemic COVID-19, appropriate when dexamethasone-equivalent doses of 6 mg/day were used for 10 days, or indicated but inappropriate when dexamethasone-equivalent doses >6 mg/day were used for >10 days. To treat mucormycosis, patients received liposomal amphotericin B (5 mg/kg 1×/d for 4–6 weeks, or, if the patient had economic constraints, amphotericin B deoxycholate 1 mg/kg 1×/d for 6–8 weeks). Duration of induction therapy was dependent on how well patients tolerated amphotericin B infusion. Oral triazoles were given for variable duration depending on the site of mucormycosis, radiologic resolution, and clinical response. Patients with intracranial extension received higher doses of amphotericin B for longer periods. We classified antifungal therapy as combination when the patient received both classes of antifungals in any formulation of amphotericin B and posaconazole or isavuconazole, concurrent when both amphotericin B and triazoles were used simultaneously, and sequential when triazole was used after amphotericin B.

### Study Objectives 

Our primary objective was to compare the epidemiology of mucormycosis between CAM and non-CAM groups during the study period, including the prevalence, underlying diseases, relationship to COVID-19, site of infection, and outcomes. Our secondary objectives were to compare CAM versus non-CAM and ascertain whether COVID-19 is a risk factor for mucormycosis death.

### Sample Processing 

Tissue biopsies from mucormycosis-affected anatomical sites were used for conventional microscopy, culture, and histopathology, as appropriate, at the respective health centers. Microscopy was performed by using potassium hydroxide mount with or without calcofluor stain. The samples were inoculated on 2 sets of Sabouraud dextrose agar and incubated at 25°C and 37°C. Positive cultures were identified by macroscopic and microscopic characteristics. Tissue samples submitted for histopathology were examined by using hematoxylin and eosin, periodic acid Schiff, or Gomori methenamine silver stain.

### Statistical Methods 

We performed data analysis using SPSS Statistics 21.0 (IBM, Inc, https://www.ibm.com). We provide descriptive statistics as frequencies, mean (SD), or median (interquartile range [IQR]), as appropriate. We compared categorical variables by using χ^2^ or Fischer exact test and analyzed differences between continuous data by using Mann-Whitney U tests. We performed multivariate logistic regression analyses to identify factors predicting development of late CAM and mucormycosis mortality rates. We considered p<0.05 statistically significant.

## Results

During the study period, a total of 295 consecutive mucormycosis cases were diagnosed at the 16 participating centers. We excluded 8 cases because of incomplete data. Of the remaining 287 cases, 187 (65.2%) had CAM. The mean age of the entire study population was 53.4 years (SD 17.1 years); 74.6% were men and 25.4% were women (Table 1). Patients with CAM were older (mean age 56.9 years), and a higher proportion (80.2%) were men than for the non-CAM patients.

**Table 1:** Baseline characteristics among patients with mucormycosis, with and without COVID-19, India*

Variables	CAM, n = 187	Non-CAM, n = 100	p value
Mean age, y (SD)	56.9 (12.5)	46.9 (16.4)	0.0001
Sex			0.003
M	150 (80.2)	64 (64.0)	
F	37 (19.8)	36 (36.0)	
Underlying disease			0.0001
None	0	19 (19.0)	
COVID-19 only	61 (32.6)	0	
Glucocorticoids for COVID-19	48/61 (78.7)	NA	
Diabetes mellitus	113 (60.4)	67 (67.0)	
Traumatic inoculation (dental surgery, trauma, and burns)	3 (1.6)	9 (9.0)	
Hematological malignancy	2 (1.1)	2 (2)	
Renal transplantation	3 (1.6)	0	
Other†	5 (2.7)	3 (3)	
Glucocorticoids	146 (78.1)	6 (6.0)	0.0001
Site of involvement			
Rhino-orbital	117 (62.6)	50 (50.0)	0.07
Rhino-orbito-cerebral	44 (23.5)	34 (34.0)	0.07
Pulmonary	16 (8.6)	6 (6.0)	0.42
Renal	1 (0.5)	1 (1.0)	0.66
Other (e.g., cutaneous, stomach)	5 (2.7)	9 (9.0)	0.03
Disseminated	4 (2.1)	0	0.41
Microscopy			0.10
Negative smear	30 (16.0)	10 (10.0)	
Aseptate hyphae	153 (81.8)	84 (84.0)	
Septate hyphae	1 (0.5)	0	
Septate and aseptate hyphae	3 (1.6)	6 (6.0)	
Culture			0.04
No growth	87 (46.5)	61 (61.0)	
* Mucorales*	99 (52.9)	37 (37.0)	
*Mucorales* and *Aspergillus* species	1 (0.5)	1 (1.0)	
*Aspergillus* species	0	1 (1.0)	
Histopathology diagnostic of mucormycosis‡	143/155 (92.3)	37/44 (84.1)	0.10
Management and outcome			
Hypoxemia during hospitalization	74 (39.6)	12 (12.0)	0.0001
Admission to the intensive care unit	58 (31.0)	9 (9.0)	0.0001
Treatment			
Liposomal amphotericin B	136 (72.7)	84 (84)	0.002
Amphotericin D deoxycholate	31 (16.6)	5 (5.0)	0.005
Posaconazole	73 (39.0)	14 (14.0)	0.0001
Isavuconazole	19 (10.2)	2 (2.0)	0.01
Combined antifungal therapy			0.0001
Single antifungal drug	95 (50.8)	88 (88.0)	
Concurrent	13 (7.0)	1 (1.0)	
Sequential	79 (42.5)	11 (11.0)	
Combined medical and surgical therapy	131 (70.1)	73 (73.0)	0.60
Outcome			
Death <6 weeks	70 (37.4)	40 (40.0)	0.67
Death <12 weeks (n = 256)	75/170 (44.1)	42/86 (48.8)	0.51

*Values are no. (%) except as indicated. CAM, COVID-19–associated mucormycosis; COVID-19, coronavirus disease; NA, not applicable. †Includes liver cirrhosis, immunosuppression, and malignancies. ‡Histopathological examination was performed in 199 cases, 155 in the CAM group and 44 non-CAM groups.

### CAM Prevalence

**Figure 2:**
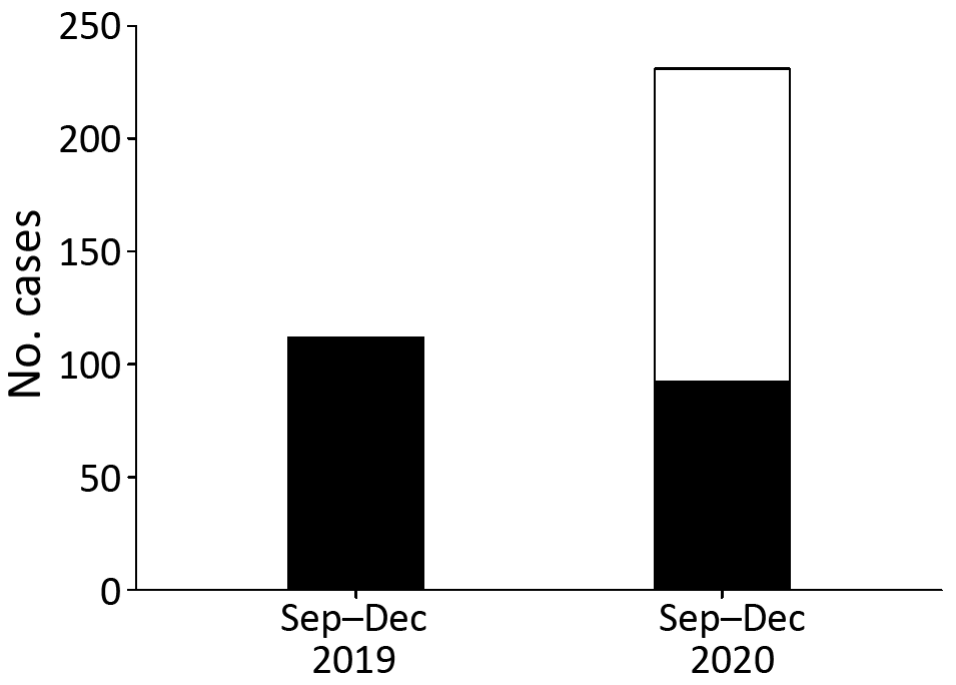
Cumulative number of mucormycosis cases during September–December 2019 and September–December 2020 in 10 health centers, India. White bar section indicates coronavirus disease–associated mucormycosis (CAM); black bar sections indicate non-CAM cases. During 2019, 112 cases of mucormycosis were detected, but a total of 231 cases, 92 non-CAM and 139 CAM, were detected in 2020.

Among participating centers, 7 provided information needed to estimate the prevalence of CAM. During the study period, CAM patients accounted for 28/10,517 COVID-19 patients managed in general wards and 25/1,579 in ICUs. The overall prevalence of CAM was 0.27% (range 0.05%–0.57%); prevalence of CAM in ICUs was 1.6% (range 0.65%–2.0%). More mucormycosis cases were identified during the 2020 study period (231 cases) than during the same time range in 2019 (112 cases). The number of mucormycosis cases unrelated to COVID-19 did not differ much during both the study periods (112 cases in 2019 vs. 92 cases in 2020), indicating the increase in 2020 was chiefly attributed to CAM (Figure 2).

### Predisposing Factors 

The most common underlying disease among both CAM and non-CAM groups was uncontrolled diabetes mellitus (62.7%). Of note, newly detected diabetes mellitus was more frequent during the evaluation of mucormycosis among CAM (39/187 [20.9%]) than non-CAM (10/100 [10%]; p = 0.02) patients. Diabetic ketoacidosis was seen less often in CAM patients (16/187 [8.6%]) than in non-CAM patients (27/100 [27%]; p = 0.0001). COVID-19 was the only underlying disease in 61/187 (32.6%) CAM patients, among whom 48 (78.7%) received glucocorticoid treatment for COVID-19 management. Other risk factors, including hematologic malignancy and solid organ transplantation, were noted in few among the study population (Table 1).

### Clinical Manifestations and Site of Involvement

**Figure 3:**
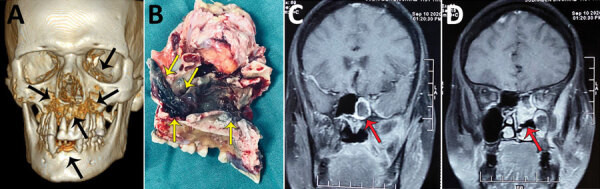
Radiographic images and surgical specimens demonstrating rhino-orbital-cerebral coronavirus disease–associated mucormycosis in patients from India, 2020. A) Three-dimensional reconstruction of computed tomography scan of 54-year-old male patient. Black arrows indicate patchy osteonecrosis involving the upper jaw, right orbital wall, and paranasal sinuses. B) Surgical specimen from the maxilla of 54-year-old male patient showing black necrotic paranasal sinus with palatal involvement indicated by yellow arrows. C, D) Magnetic resonance imaging (MRI) of coronal section of paranasal sinus and brain of 51-year-old female patient. Red arrow in panel C indicates enhancing cavernous sinus lesion; D) red arrow in panel D indicates right ethmoid and maxillary sinusitis. Scale bar indicates 7 cm.

**Figure 4:**
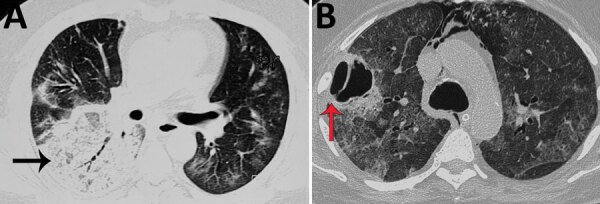
Noncontrast computed tomography scan of the thorax of a patient with coronavirus disease–associated mucormycosis, India, 2020. A) Pulmonary mucormycosis demonstrated as a large area of consolidations with patchy air trapping (black arrow), patchy ground-glass opacities, and septal thickening; B) large thick-walled cavity (red arrow) with surrounding ground-glass opacities.

A greater percentage of patients with CAM had hypoxemia requiring ICU admission during hospitalization than the non-CAM group (Table 1). The rhino-orbital region was the most common mucormycosis site (58.2%), followed by rhino-orbital-cerebral, pulmonary, and other sites (Table 1). However, site of involvement was similar in both the CAM and the non-CAM groups. Toothache, loosening of teeth, and radiologic involvement of the jaw were noted in many CAM patients (Figure 3) but were not seen in non-CAM patients. One participating center reported jaw involvement in 10/47 (21.3%) contributed CAM cases (Figure 3). The common form of pulmonary involvement was cavitary lung disease (Figure 4).

### Diagnosis 

Mucormycosis diagnosis was made by direct microscopy in 237/287 (82.6%) patients. Histopathology demonstrated aseptate hyphae in 180/199 (90.5%) patients. Culture identified the etiologic agent in 138/287 (48.1%) cases (Table 1). The isolated Mucorales included *Rhizopus arrhizus*, *Rhizomucor pusillus*, *Apophysomyces variabilis*, *Lichtheimia corymbifera*, and others. We did not note association of any species with any anatomic infection site.

### Treatment 

Liposomal amphotericin B was the most used antifungal agent in both groups. However, the use of liposomal amphotericin B was much lower in the CAM group (72.7%) compared with the non-CAM group (84%). Posaconazole and isavuconazole were more frequently used in CAM patients than in the non-CAM group. A combination of antifungal therapy, such as amphotericin B plus triazoles, either concurrent or sequential, was used much more often in CAM patients (49.5%) than in non-CAM (12%) patients. Combined medical and surgical management was performed in 71.1% (204/287) of patients and was similar in the 2 groups. Major resection of the affected site was performed in 59/284 patients; the remaining patients underwent partial resection or debridement.

### Outcomes 

Mortality rates were similar between CAM and non-CAM groups; the combined 6-week mortality rate was 38.3% (110/287 patients) and the 12-week mortality rate was 45.7% (117/256 patients) (Table 1). Univariate analysis showed that combined medical and surgical management improved survival in the rhino-orbital-cerebral group but did not improve outcomes for patients with infections at other sites (Appendix Table 1). On multivariate logistic regression analysis, we found age, site of involvement (rhino-orbital-cerebral or pulmonary), and ICU admission were associated with increased mortality rates. In contrast, sequential treatment with a combination of antifungal drugs was independently associated with better survival at 6 and 12 weeks (Table 2; Appendix Table 2).

**Table 2:** Multivariate analysis of factors predicting death at 6 weeks among patients with mucormycosis, India*

Variables	Survivors, n = 177	Non-survivors, n = 110	Odds ratio (95% CI)	p value
Mean age, y (SD)	52.6 (15.1)	54.7 (14.0)	1.02 (1.00–1.04)	**0.03**
Underlying disease				
None	10 (5.6)	9 (8.2)	Referent	Referent
Isolated COVID-19	42 (23.7)	19 (17.3)	0.56 (0.17–1.83)	0.34
Diabetes mellitus	109 (61.6)	71 (64.5)	0.92 (0.32–2.64)	0.88
Traumatic inoculation	8 (4.5)	4 (3.6)	1.30 (0.25–6.80)	0.76
Others	5 (2.8)	3 (2.7)	1.20 (0.18–7.81)	0.85
Renal transplantation	1 (0.6)	2 (1.8)	6.87 (0.42–113.19)	0.18
Hematological malignancy	2 (1.1)	2 (1.8)	1.60 (0.14–18.72)	0.71
Site of involvement				
Rhino-orbital	117 (66.1)	50 (45.5)	Referent	Referent
Rhino-orbito-cerebral	39 (22)	39 (35.5)	2.39 (1.30–4.40)	**0.005**
Pulmonary	8 (4.5)	14 (12.7)	3.26 (1.05–10.11)	**0.04**
Other†	13 (7.3)	7 (6.4)	1.29 (0.43–3.86)	0.64
Admission to the intensive care unit	32 (18.1)	35 (31.8)	2.87 (1.43–5.75)	**0.003**
Combined medical surgical therapy	135 (76.3)	69 (62.7)	0.77 (0.41–1.45)	0.41
Combination of antifungals				
Single antifungal drug	95 (53.7)	88 (80)	Referent	Referent
Concurrent	9 (5.1)	5 (4.5)	0.37 (0.09–1.44)	0.15
Sequential	73 (41.2)	17 (15.5)	0.17 (0.87–0.35)	**0.0001**

*Values are no. (%) except as indicated. Bold text indicates statistical significance. COVID-19, coronavirus disease. †Includes cutaneous, stomach, disseminated, or other.

### Subgroup Analysis of CAM 

**Figure 5:**
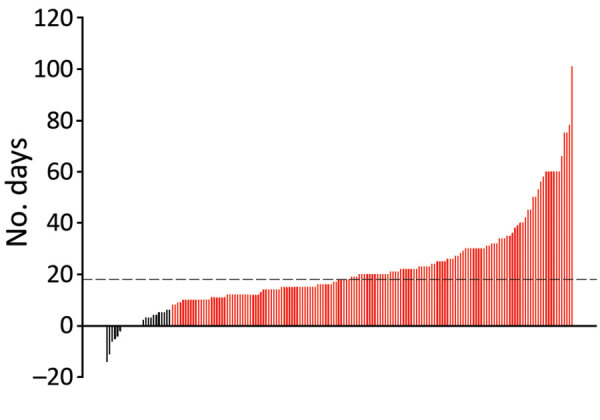
Waterfall plot showing the number of days between the diagnosis of coronavirus disease (COVID-19) and COVID-19–associated mucormycosis (CAM). Each vertical line represents a case-patient. Red indicates late CAM (mucormycosis developing >8 days after COVID-19 diagnosis); black indicates early CAM (mucormycosis developing <7 days of COVID-19 diagnosis). Among early CAM cases, mucormycosis was diagnosed before (n = 8), concurrently with (n = 8), or after (n = 13) COVID-19 diagnosis. Dotted line represents the median duration (18 days) after COVID-19 diagnosis for the diagnosis of CAM.

The median time to CAM diagnosis was 18 (IQR 11–27) days (Figure 5). Among 187 CAM patients, 158 (84.2%) were classified as late CAM (Table 3). Some (33/187; 17.6%) patients were managed for COVID-19 at home before developing CAM. Among 187 CAM patients, 74 (55.6%) were hypoxemic. Glucocorticoid drugs were administered in various doses; the median cumulative dexamethasone-equivalent dose was 84 mg (range 18–1,343 mg). Of note, only 49/146 (33.6%) patients received steroids at appropriate levels (Table 3). Tocilizumab was administered to 5 (2.7%) patients for COVID-19 management.

**Table 3:** Characteristics of early and late CAM among patients with COVID-19, India*

Variables	Early CAM, n = 29†	Late CAM, n = 158‡	p value
Mean age, y (SD)	51.8 (14.2)	57.8 (11.9)	0.015
Sex			0.10
F	9 (31.0)	28 (17.7)	
M	20 (69.0)	130 (82.3)	
Glucocorticoids	8 (27.6)	138 (87.3)	0.0001
Tocilizumab	0	5 (3.2)	0.33
Underlying diseases			0.52
COVID-19 only	11 (37.9)	50 (31.6)	
Diabetes mellitus	16 (55.2)	97 (61.4)	
Diagnosed during current illness	6	33	
Diabetic ketoacidosis§	8	8	
Traumatic inoculation: dental surgery, trauma, and burns	0	3 (1.9)	
Hematological malignancy	0	2 (1.3)	
Renal transplantation	0	3 (1.9)	
Other: liver cirrhosis, immunosuppression, and others	2 (6.9)	3 (1.9)	
Site of involvement			0.88
Rhino-orbital	17 (58.7)	100 (63.3)	
Rhino-orbito-cerebral	8 (27.6)	36 (22.8)	
Pulmonary	3 (10.3)	13 (8.2)	
Renal	0	1 (0.6)	
Other: e.g., cutaneous, stomach	0	5 (3.2)	
Disseminated	1 (3.4)	3 (1.9)	
Hypoxemia during hospitalization	9 (31.0)	65 (41.1)	0.19
ICU admission	12 (41.4)	46 (29.1)	0.31
Glucocorticoid treatment for COVID-19	N = 17	N = 133	
Appropriate	11 (64.7)	44 (33.1)	
Not indicated	4 (23.5)	46 (34.6)	
Indicated, but inappropriately high dose	2 (11.8)	43 (32.3)	
Treatment			
Liposomal amphotericin B	26 (89.7)	110 (71.9)	0.06
Amphotericin D deoxycholate	3 (10.3)	28 (17.7)	0.33
Posaconazole	4 (13.8)	69 (43.7)	0.02
Isavuconazole	0	19 (12.0)	0.049
Combined antifungal therapy			0.004
Single antifungal drug	23 (79.4)	72 (45.6)	
Concurrent	1 (3.4)	12 (7.6)	
Sequential	5 (17.2)	74 (46.8)	
Combined medical and surgical therapy	18 (62.1)	113 (71.5)	0.31
Outcomes			
Death at 6 weeks	12 (41.4)	58 (36.7)	0.63
Death at 12 weeks, n = 170	13/22 (59.1)	62/148 (41.9)	0.17


*Values are no. (%) except as indicated. CAM, COVID-19–associated mucormycosis; COVID-19, coronavirus disease; ICU, intensive care unit. †Early CAM is considered mucormycosis diagnosed <7 days of COVID-19 diagnosis. ‡Late CAM is mucormycosis diagnosed >8 days of COVID-19 diagnosis. §Diabetic ketoacidosis was more frequent among patients with early CAM (p = 0.0001).

The demographic characteristics, underlying diseases, and site of involvement were similar among patients with early and late CAM. However, we saw diabetic ketoacidosis more often in patients with early CAM (28%) than late CAM (5%). A higher proportion of patients with late CAM received glucocorticoid treatment (Table 3). Whereas amphotericin B remained the most common antifungal drugs used in both groups, posaconazole, isavuconazole, or a sequential use of antifungal agents (i.e., amphotericin B followed by posaconazole or isavuconazole) was more often seen in patients with late CAM. We saw no statistically significant difference in 6- and 12-week mortality rates between the early and late CAM groups (Table 3).

We also explored factors associated with late CAM development (Table 4). After adjusting for age, sex, and underlying risk factors, we found hypoxemia due to COVID-19 and inappropriate glucocorticoid administration were associated with development of late CAM.

**Table 4:** Multivariate analysis of factors predicting the development of late CAM among COVID-19 patients, India*

Variables	Early CAM, n = 29†	Late CAM, n = 158‡	Odds ratio (95% CI)	p value
Mean age, y (SD)	51.8 (14.2)	57.8 (11.9)	1.02 (0.96–1.07)	0.62
Sex				
M	20 (69.0)	130 (82.3)	0.25 (0.06–1.10)	0.07
F	9 (31.0)	28 (17.7)	Referent	
Underlying disease				
Isolated COVID-19	11 (23.7)	50 (17.3)	1.71 (0.25–11.96)	0.59
Diabetes mellitus	16 (61.6)	97 (64.5)	5.84 (0.70–48.89)	0.10
Others§	2 (4.5)	11 (3.6)	Referent	
Hypoxemia due to COVID-19	9 (31.0)	65 (41.1)	11.84 (1.43–98.06)	**0.02**
Glucocorticoid usage	N = 17	N = 133		
Appropriate	11 (64.7)	44 (33.1)	Referent	
Not indicated	4 (23.5)	46 (34.6)	66.93 (7.05–635.19)	**0.0001**
Indicated, but inappropriately high dose	2 (11.8)	43 (32.3)	9.91 (1.39–70.77)	**0.02**

*Values are no. (%) except as indicated. Bold text indicates statistical significance. CAM, COVID-19–associated mucormycosis; COVID-19, coronavirus disease. †Early CAM is considered mucormycosis diagnosed <7 days of COVID-19 diagnosis. ‡Late CAM is mucormycosis diagnosed >8 days of COVID-19 diagnosis. §Includes traumatic inoculation, cirrhosis, immunosuppression, renal transplantation, and hematological malignancy

## Discussion

In our study, the prevalence of CAM was 0.27% in patients managed in hospital wards and 1.6% in patients managed in ICUs. We found a 2.1-fold increase in mucormycosis cases during September–December 2020 than the same months of 2019; we attribute the increase to COVID-19. Most CAM cases were diagnosed >8 days after COVID-19 diagnoses. Hypoxemia due to COVID-19 and inappropriate use of glucocorticoid drugs were independently associated with development of late CAM. The mortality rate for CAM patients was high (44%) but was comparable to rates for non-CAM (49%) patients. Older age (>54 years), admission to an ICU, and pulmonary or brain involvement by Mucorales were independently associated with a higher risk for death. The sequential use of antifungal drugs at any site was associated with improved survival at 6 and 12 weeks, irrespective of anatomical site of mucormycosis.

In our study, 74.6% of patients affected by mucormycosis were men, as observed in previous studies (*11*–*13*). We found diabetes mellitus was the most common underlying disease for both CAM and non-CAM patients. SARS-CoV-2 has been shown to affect the beta cells of the pancreas, resulting in metabolic derangement, possibly causing diabetes mellitus (*14*,*15*). Whether more frequent diagnosis (20%) of diabetes mellitus during the evaluation for CAM compared with non-CAM (10%) is related to SARS-CoV-2 infection, glucocorticoid therapy, or a chance occurrence remains unclear. Unfortunately, we do not have glycated hemoglobin values taken at admission for all newly detected diabetes cases in our study, so we cannot determine if these patients had diabetes mellitus before CAM developed. 

We found inappropriate glucocorticoid use was independently associated with late CAM. Among 187 CAM cases, 61 (32.6%) had COVID-19 as the only underlying disease; 13 of those cases were not treated with glucocorticoid or other immunomodulatory therapies. Whether COVID-19 itself causes immune dysregulation and predisposes patients to invasive mucormycosis remains an unproven possibility (*16*–*18*). We did not find that COVID-19 was an independent predictor of late CAM, possibly because of the lower numbers of patients in our cohort with COVID-19 as the only underlying disease without any other risk factor. Lymphopenia is common in COVID-19, and progressive lymphopenia has been shown to correlate with COVID-19 severity (*19*). The persisting immune dysregulation during the recovery phase of COVID-19 infection also confers additional risk. Unfortunately, we have not evaluated the effect of lymphopenia on the development or outcome of CAM. Tocilizumab use in COVID-19 has been reported as a risk factor for invasive candidiasis (*20*). However, only 2.7% of the CAM patients in this study received tocilizumab.

The high mortality rate for CAM is a major concern (*7*). Patients with CAM were older (56.9 years) than non-CAM patients (46.9 years). Evidence suggests that older age imparts increased risk for hospitalization, respiratory failure, ICU admission, and attendant glucocorticoid therapy in COVID-19 (*21*,*22*). Further, age >54 years also was associated with an increased risk for death among our cohort. The site of mucormycosis involvement and the survival at 6 and 12 weeks was similar in CAM and non-CAM groups. We expected a higher proportion of pulmonary mycosis because respiratory viral infections, such as influenza, often are associated with secondary invasive aspergillosis (*8*). However, we did not observe an increased occurrence of pulmonary mucormycosis compared with infections in other sites among the CAM group. Considering the low rate of pulmonary involvement, we believe that CAM can be attributed to the systemic effects of COVID-19 or its treatment, rather than a sole alteration in the lungs. Several pulmonary mucormycosis cases also might have remained undiagnosed because of challenges in obtaining diagnostic respiratory samples among critically ill COVID-19 patients.

Appropriate and timely antifungal therapy and surgical resection, when feasible, are considered essential in mucormycosis management. Liposomal amphotericin B is the drug of choice, but isavuconazole also is recommended in primary therapy. Triazoles, including posaconazole and isavuconazole, commonly are used in the consolidation phase or as salvage therapy (*23*). The role of combination antifungal treatment in mucormycosis is not clearly supported by evidence (*24*). The combination of surgery and antifungal therapy was associated with better survival in the rhino-orbital-cerebral group in this study, conforming with previous experiences (*6*,*11*,*25*). However, the same was not true for mucormycosis in other anatomic sites. Early diagnosis of mucormycosis and the more frequent use of consolidation therapy or combination of antifungals in this study could be one explanation; another could be fewer surgeries performed in patients with other than rhino-orbital mucormycosis.

We found the sequential use of antifungal drugs, amphotericin B then posaconazole or isavuconazole, was independently associated with improved survival among mucormycosis patients. However, the lack of randomization, possibility of case selection, and chance survival are potential biases. In addition, the optimal duration and dose of amphotericin B and posaconazole are not clear. The usefulness of antifungal combination administered simultaneously could not be ascertained due to the small number of patients receiving concurrent therapy in our study. A randomized controlled trial could affirm the role of a combination of antifungals or maintenance therapy in mucormycosis.

We expected better survival for the CAM patients in this study. Contrary to the prevailing practices (*11*,*24*), a combination of antifungal agents was more frequently used (50%) in CAM patients than in non-CAM patients (12%). Also, hospitalized CAM patients were closely monitored. The treatment practices used for the CAM group, especially those with late CAM, were distinct from those for the non-CAM group and those for patients with early CAM. The occurrence of a mold infection and the apprehension associated with the COVID-19 pandemic could have resulted in more frequent use of combination therapy in CAM. However, we saw no difference in mortality rates between CAM and non-CAM patients. Of course, increased risk for death due to COVID-19 itself cannot be ruled out for these CAM patients.

Our study’s first limitation is that we collected data from a single country. The predominant risk factor for mucormycosis in our study was diabetes, which is also the case in some countries, including Bangladesh, China, Iran, Mexico, and Pakistan, from which data on mucormycosis are still limited (*26*). Further studies should compare data from countries with high rates of diabetes and mucormycosis with that of data from the United States and Europe, where mucormycosis predominantly is encountered in hematological malignancies and organ transplantation. Given the large number of late CAM cases, healthcare-associated mucormycosis remains a distinct possibility (*27*,*28*). Contaminated ventilation systems, air conditioners, and ongoing construction in hospitals have been reported to cause outbreaks of mucormycosis in the past (*28*). However, we did not estimate the burden of Mucormycetes spores in the hospital environment (*29*). We also do not have data on the timing of amphotericin B use, timing of surgery, or duration of sequential antifungal therapy, which are critical factors that have a bearing on mucormycosis outcomes; hence, we could not analyze these factors. Other unexplored factors, including genetic predisposition, might explain the high prevalence of CAM and non-CAM in India. Thus, prospective studies from the rest of the world, especially those severely affected by the COVID-19 pandemic, would be needed to ascertain the epidemiology of CAM. The strength of our study is the large number of patients, which lends credibility to our observations.

In conclusion, mucormycosis is a rare but critical problem complicating the later part of the clinical course of COVID-19 in India, possibly due to improper glucocorticoid usage. We found no difference in the risk factors, site of involvement, and outcome of mucormycosis complicating COVID-19 cases compared with non–COVID-19 cases. Nevertheless, the prevalence of mucormycosis has increased greatly in India, coinciding with the country’s COVID-19 epidemic. Clinicians should be vigilant for mucormycosis in the patients recovering from COVID-19 illness, especially among patients with new or previously diagnosed diabetes mellitus and clinical manifestations of facial or orbital pain or black or blood-stained nasal discharge. In addition, we found improper glucocorticoid use for the COVID-19 treatment to be an additional risk factor in CAM. Therefore, treating physicians should ensure they use appropriate drugs and doses in treating COVID-19 patients.

Appendix Additional information on COVID-19–associated mucormycosis, India, 2020.

## References

[1] Baiou A, Elbuzidi AA, Bakdach D, Zaqout A, Alarbi KM, Bintaher AA, et al. Clinical characteristics and risk factors for the isolation of multi-drug-resistant Gram-negative bacteria from critically ill patients with COVID-19. J Hosp Infect. 2021;110:165–71. 10.1016/j.jhin.2021.01.027 33561503 PMC7866848

[2] Ripa M, Galli L, Poli A, Oltolini C, Spagnuolo V, Mastrangelo A, et al.; COVID-BioB study group. Secondary infections in patients hospitalized with COVID-19: incidence and predictive factors. Clin Microbiol Infect. 2021;27:451–7. 10.1016/j.cmi.2020.10.021 33223114 PMC7584496

[3] Seaton RA, Gibbons CL, Cooper L, Malcolm W, McKinney R, Dundas S, et al. Survey of antibiotic and antifungal prescribing in patients with suspected and confirmed COVID-19 in Scottish hospitals. J Infect. 2020;81:952–60. 10.1016/j.jinf.2020.09.024 32987097 PMC7518971

[4] Nucci M, Barreiros G, Guimarães LF, Deriquehem VAS, Castiñeiras AC, Nouér SA. Increased incidence of candidemia in a tertiary care hospital with the COVID-19 pandemic. Mycoses. 2021;64:152–6. 10.1111/myc.13225 33275821 PMC7753494

[5] van Arkel ALE, Rijpstra TA, Belderbos HNA, van Wijngaarden P, Verweij PE, Bentvelsen RG. COVID-19–associated pulmonary aspergillosis. Am J Respir Crit Care Med. 2020;202:132–5. 10.1164/rccm.202004-1038LE 32396381 PMC7328331

[6] Chong WH, Saha BK, Ananthakrishnan Ramani, Chopra A. Ananthakrishnan Ramani, Chopra A. State-of-the-art review of secondary pulmonary infections in patients with COVID-19 pneumonia. Infection. 2021; [Epub ahead of print]. 10.1007/s15010-021-01602-z PMC7951131 33709380

[7] Garg D, Muthu V, Sehgal IS, Ramachandran R, Kaur H, Bhalla A, et al. Coronavirus disease (Covid-19) associated mucormycosis (CAM): case report and systematic review of literature. Mycopathologia. 2021;186:289–98. 33544266 10.1007/s11046-021-00528-2 PMC7862973

[8] Ahmadikia K, Hashemi SJ, Khodavaisy S, Getso MI, Alijani N, Badali H, et al. The double-edged sword of systemic corticosteroid therapy in viral pneumonia: A case report and comparative review of influenza-associated mucormycosis versus COVID-19 associated mucormycosis. Mycoses. 2021;myc.13256; Epub ahead of print. 10.1111/myc.13256 33590551 PMC8013756

[9] Moorthy A, Gaikwad R, Krishna S, Hegde R, Tripathi KK, Kale PG, et al. SARS-CoV-2, uncontrolled diabetes and corticosteroids—an unholy trinity in invasive fungal infections of the maxillofacial region? A retrospective, multi-centric analysis. J Maxillofac Oral Surg. 2021;1–8; [Epub ahead of print]. 33716414 10.1007/s12663-021-01532-1 PMC7936599

[10] Joshi SR, Das AK, Vijay VJ, Mohan V. Challenges in diabetes care in India: sheer numbers, lack of awareness and inadequate control. J Assoc Physicians India. 2008;56:443–50. 18822625

[11] Patel A, Kaur H, Xess I, Michael JS, Savio J, Rudramurthy S, et al. A multicentre observational study on the epidemiology, risk factors, management and outcomes of mucormycosis in India. Clin Microbiol Infect. 2020;26:944.e9–15. 10.1016/j.cmi.2019.11.021 31811914

[12] Jeong W, Keighley C, Wolfe R, Lee WL, Slavin MA, Kong DCM, et al. The epidemiology and clinical manifestations of mucormycosis: a systematic review and meta-analysis of case reports. Clin Microbiol Infect. 2019;25:26–34. 10.1016/j.cmi.2018.07.011 30036666

[13] Prakash H, Ghosh AK, Rudramurthy SM, Singh P, Xess I, Savio J, et al. A prospective multicenter study on mucormycosis in India: Epidemiology, diagnosis, and treatment. Med Mycol. 2019;57:395–402. 10.1093/mmy/myy060 30085158

[14] Müller JA, Groß R, Conzelmann C, Krüger J, Merle U, Steinhart J, et al. SARS-CoV-2 infects and replicates in cells of the human endocrine and exocrine pancreas. Nat Metab. 2021;3:149–65. 10.1038/s42255-021-00347-1 33536639

[15] Accili D. Can COVID-19 cause diabetes? Nat Metab. 2021;3:123–5. 10.1038/s42255-020-00339-7 33432203 PMC8892570

[16] Files JK, Boppana S, Perez MD, Sarkar S, Lowman KE, Qin K, et al. Sustained cellular immune dysregulation in individuals recovering from SARS-CoV-2 infection. J Clin Invest. 2021;131:e140491. 10.1172/JCI140491 33119547 PMC7773371

[17] Potenza L, Vallerini D, Barozzi P, Riva G, Forghieri F, Zanetti E, et al. Mucorales-specific T cells emerge in the course of invasive mucormycosis and may be used as a surrogate diagnostic marker in high-risk patients. Blood. 2011;118:5416–9. 10.1182/blood-2011-07-366526 21931119

[18] Ghuman H, Voelz K. Innate and adaptive immunity to mucorales. J Fungi (Basel). 2017;3:48. 10.3390/jof3030048 29371565 PMC5715954

[19] Zhang X, Tan Y, Ling Y, Lu G, Liu F, Yi Z, et al. Viral and host factors related to the clinical outcome of COVID-19. Nature. 2020;583:437–40. 10.1038/s41586-020-2355-0 32434211

[20] Antinori S, Bonazzetti C, Gubertini G, Capetti A, Pagani C, Morena V, et al. Tocilizumab for cytokine storm syndrome in COVID-19 pneumonia: an increased risk for candidemia? Autoimmun Rev. 2020;19:102564. 10.1016/j.autrev.2020.102564 32376396 PMC7200127

[21] Levin AT, Hanage WP, Owusu-Boaitey N, Cochran KB, Walsh SP, Meyerowitz-Katz G. Assessing the age specificity of infection fatality rates for COVID-19: systematic review, meta-analysis, and public policy implications. Eur J Epidemiol. 2020;35:1123–38. 10.1007/s10654-020-00698-1 33289900 PMC7721859

[22] Pijls BG, Jolani S, Atherley A, Derckx RT, Dijkstra JIR, Franssen GHL, et al. Demographic risk factors for COVID-19 infection, severity, ICU admission and death: a meta-analysis of 59 studies. BMJ Open. 2021;11:e044640. 10.1136/bmjopen-2020-044640 33431495 PMC7802392

[23] Cornely OA, Alastruey-Izquierdo A, Arenz D, Chen SCA, Dannaoui E, Hochhegger B, et al.; Mucormycosis ECMM MSG Global Guideline Writing Group. Global guideline for the diagnosis and management of mucormycosis: an initiative of the European Confederation of Medical Mycology in cooperation with the Mycoses Study Group Education and Research Consortium. Lancet Infect Dis. 2019;19:e405–21. 10.1016/S1473-3099(19)30312-3 31699664 PMC8559573

[24] Jeong W, Keighley C, Wolfe R, Lee WL, Slavin MA, Chen SC, et al. Contemporary management and clinical outcomes of mucormycosis: A systematic review and meta-analysis of case reports. Int J Antimicrob Agents. 2019;53:589–97. 10.1016/j.ijantimicag.2019.01.002 30639526

[25] Muthu V, Agarwal R, Dhooria S, Sehgal IS, Prasad KT, Aggarwal AN, et al. Has the mortality from pulmonary mucormycosis changed over time? A systematic review and meta-analysis. Clin Microbiol Infect. 2021;27:538–49. 10.1016/j.cmi.2020.12.035 33418022

[26] Prakash H, Chakrabarti A. Global Epidemiology of mucormycosis. J Fungi (Basel). 2019;5:26. 10.3390/jof5010026 30901907 PMC6462913

[27] Rammaert B, Lanternier F, Zahar JR, Dannaoui E, Bougnoux ME, Lecuit M, et al. Healthcare-associated mucormycosis. Clin Infect Dis. 2012;54(Suppl 1):S44–54. 10.1093/cid/cir867 22247444

[28] Walther G, Wagner L, Kurzai O. Outbreaks of mucorales and the species involved. Mycopathologia. 2020;185:765–81. 31734800 10.1007/s11046-019-00403-1

[29] Prakash H, Singh S, Rudramurthy SM, Singh P, Mehta N, Shaw D, et al. An aero mycological analysis of *Mucormycetes* in indoor and outdoor environments of northern India. Med Mycol. 2020;58:118–23. 10.1093/mmy/myz031 30980083

