# Supplementary material for: Multicenter Epidemiologic Study of Coronavirus Disease–Associated Mucormycosis, India
Source: Emerg Infect Dis. 2021 Sep;27(9):2349–59. doi: 10.3201/eid2709.210934 (PMC8386807; doi:10.3201/eid2709.210934)
Supplement: Appendix — Additional information on COVID-19–associated mucormycosis, India, 2020. [file 21-0934-Techapp-s1.pdf]

Article DOI: https://doi.org/10.3201/eid2709.210934

Multicenter Epidemiologic Study of COVID-19–Associated Mucormycosis, India

Appendix

Appendix Table 1. Mortality rates a 6 and 12 weeks among patients treated with combined surgical and medical management for mucormycosis, India*

Site of mucormycosis	Alive, no. (%)	Dead, no. (%)	p value
6-Week mortality rates			
Rhino-orbital	89/117 (76.1)	38/50 (76)	0.99
Other (cutaneous, stomach, disseminated, other)	9/13 (69.2)	6/7 (85.7)	0.61
Pulmonary	2/8 (25)	2/14 (14.3)	0.54
Rhino-orbital-cerebral	35/39 (89.7)	23/39 (59)	0.004
12-Week mortality rates			
Rhino-orbital	76/98 (77.6)	41/53 (77.4)	0.98
Other (cutaneous, stomach, disseminated, other)	7/9 (77.8)	7/8 (87.5)	0.60
Pulmonary	2/9 (22.2)	2/13 (15.4)	0.69
Rhino-orbital-cerebral	22/23 (95.7)	28/43 (65.1)	0.006

Appendix Table 2. Multivariate analysis of factors predicting death at 12 weeks in patients with mucormycosis, India*

Variables	Survived (n = 139)	Died (n = 117)	OR (95% CI)	p value
Mean age, y (SD)	51.6 (15.1)	55.5 (14.3)	1.03 (1.01–1.06)	**0.002**
Underlying disease				
None	9 (6.5)	8 (6.8)		
COVID-19	34 (24.5)	21 (17.9)	0.90 (0.26–3.10)	0.87
Diabetes mellitus	82 (59)	75 (64.2)	1.32 (0.43–4.04)	0.63
Traumatic inoculation	6 (4.3)	6 (5.1)	3.39 (0.63–18.23)	0.16
Others	5 (3.6)	3 (2.6)	1.05 (0.15–7.22)	0.96
Renal transplantation	1 (0.7)	2 (1.7)	6.98 (0.43–113.86)	0.17
Hematological malignancy	2 (1.4)	2 (1.7)	1.73 (0.13–23.38)	0.68
Site of involvement				
Rhino-orbital	98 (70.5)	53 (45.3)		
Rhino-orbital-cerebral	23 (16.5)	43 (36.8)	3.77 (1.92–7.39)	**0.0001**
Pulmonary	9 (6.5)	13 (11.1)	2.26 (0.73–7.02)	0.16
Others†	9 (6.5)	8 (6.8)	1.53 (0.48–4.91)	0.47
Admission to the intensive care unit	25 (18)	35 (29.9)	2.59 (1.24–5.42)	**0.011**
Combined medical surgical therapy	107 (77)	78 (66.7)	0.79 (0.388–1.59)	0.50
Combination of antifungals				
Single antifungal drug	70 (50.4)	88 (75.2)		
Concurrent	8 (5.7)	5 (4.3)	0.29 (0.07–1.19)	0.09
Sequential	61 (43.9)	24 (20.5)	0.20 (0.10–0.39)	**0.0001**

*Values are no. (%) except where otherwise indicated. COVID-19, coronavirus disease; OR, odds ratio. Bold text indicates statistical significance.
†Includes cutaneous, stomach, disseminated and others.
